# Evaluation of implanted perforated lacrimal punctal plugs using anterior segment optical coherence tomography

**DOI:** 10.1186/s40662-021-00259-x

**Published:** 2021-10-03

**Authors:** Raafat Mohyeldeen Abdelrahman Abdallah, Ahmed Mohamed Kamal Elshafei, Heba Radi AttaAllah

**Affiliations:** 1 Ophthalmology Department, Minia University, El-Minia, Egypt; 2 Ophthalmology Department, Minia University Hospital, El-Minia, Egypt

**Keywords:** AS-OCT, Perforated plugs, Plugs

## Abstract

**Purpose:**

Evaluation of the patency and position of perforated lacrimal punctal plugs implanted for treating punctal stenosis together with quantitative assessment of the precorneal tear film using anterior segment optical coherence tomography (AS-OCT).

**Methods:**

In a prospective study, the lower punctum of 54 eyes of 29 patients implanted with perforated punctal plugs were examined using AS-OCT during the early postoperative period. Preoperative tear meniscus height (TMH) and tear meniscus area (TMA) were evaluated. Postoperatively, the patency of the plug, its position, TMH and TMA were evaluated, and the results were correlated with postoperative epiphora. Munk scale was used for epiphora grading.

**Results:**

Using AS-OCT, 48 (88.9%) plugs were found in proper position while 6 (11.1%) were rotated. The lumen of the plugs was completely patent in 47 (87%) plugs, partially obstructed in 2 (3.7%) plugs and completely occluded in 5 (9.2%) plugs. There was a statistically significant postoperative decrease of TMH and TMA (*P* < 0.001) and postoperative epiphora Munk score (*P* < 0.001).

**Conclusion:**

AS-OCT is a valuable, reliable, and noninvasive investigative tool that can detect the proper positioning, patency, and contents of the implanted perforated lacrimal punctal plugs in addition to measurement of TMH and TMA.

*Trial registration* ClinicalTrials.gov ID: NCT04624022, https://clinicaltrials.gov/ct2/show/NCT04624022

**Supplementary Information:**

The online version contains supplementary material available at 10.1186/s40662-021-00259-x.

## Background

Acquired punctal stenosis is a frequent cause of epiphora [1]. A leading cause for acquired punctal stenosis is chronic inflammation with mononuclear cellular infiltration and associated fibrosis [2]. Treatment options include repeated mechanical and balloon dilation, different punctal snip operations, punctal punching, punctoplasty with mitomycin-C, perforated punctal plugs, mini-monoka, and self-retaining bicanalicular tubes [3–6]. Perforated punctal plugs were first introduced by Bernard et al., with a central hole of 0.6 mm made to permit drainage of tears. Due to the hydrophilic nature of its silicone material, accumulated secretions may block the lumen [7]. Coating the plugs with polyvinylpyrrolidone (PVP) makes them hydrophobic and allows tears and debris to flow smoothly through the perforation [6].

Anterior segment optical coherence tomography (AS-OCT) was recently used to provide high-resolution images of the punctum [8, 9]. Being a noncontact and noninvasive diagnostic modality, AS-OCT could be efficiently used for evaluating and measuring the lower punctum in patients with punctal stenosis [10].

In this study, the patency and position of perforated lacrimal punctal plugs implanted for treating punctal stenosis were evaluated using AS-OCT, in addition to quantitative assessment of the precorneal tear film. To the best of our knowledge, this is the first study that utilizes AS-OCT for evaluation of the implanted perforated punctal plugs.

## Methods

This was a prospective case study series (ClinicalTrials.gov ID: NCT04624022) that included 54 eyes of 29 patients implanted with perforated punctal plugs and carried out at the Ophthalmology Department of Minia University Hospital between June 2020 and January 2021. The study was approved by the local ethics committee of the Faculty of Medicine, Minia University, and adhered to the tenets of the Declaration of Helsinki. Detailed informed consent was obtained from all participants after thorough explanation of the study objective and methodology. Fifty-four eyes of 29 patients with epiphora due to acquired punctal stenosis that were managed by implantation of perforated punctal plugs were included in the study. Exclusion criteria included eyes with previous eyelid or lacrimal drainage surgery, lid malposition, ocular inflammation, or allergy.

Preoperative AS-OCT evaluation of the tear film meniscus height and area was performed the day before the procedure. Postoperative AS-OCT was done after 2 weeks, to assess the tear film meniscus height, tear meniscus area, position and patency of the plug and presence of any abnormal content.

### Technique of AS-OCT examination

Examination of the perforated punctal plug was performed using a spectral-domain OCT (RTVue Model-RT100 CAM System, Version 6.2; Optovue, Inc., Fremont, CA, USA)™ after attaching the cornea/anterior module (CAM) lens. The CAM lens is a high-magnification wide-angle lens with a 10-mm working distance, lateral resolution of 8 μm and axial resolution of 5 μm, and the scan beam had a wavelength of 840 ± 10 nm. Exposure of the plug was performed by gentle eversion of the medial part of the lower lid, so that the vertical canaliculus will be brought to be at an axial plain without exerting stretch or pressure to the lower lid. The two red external light-emitting diodes (LEDs) were approximated on each side of the lower punctum for proper illumination and imaging of the plugs. Line scan (8 mm in length and composed of 1020 A-scans/line) was selected, centered on the lower punctum in a parallel direction with mucocutaneous junction, to evaluate plug position, patency, and contents. Appropriate plug position was considered when the collar of the plug was in proper apposition on the punctal edge. Measurement of the lower tear meniscus height and tear meniscus area was done using the method of Raj et al. [11] as follows.

**Fig. 1:**
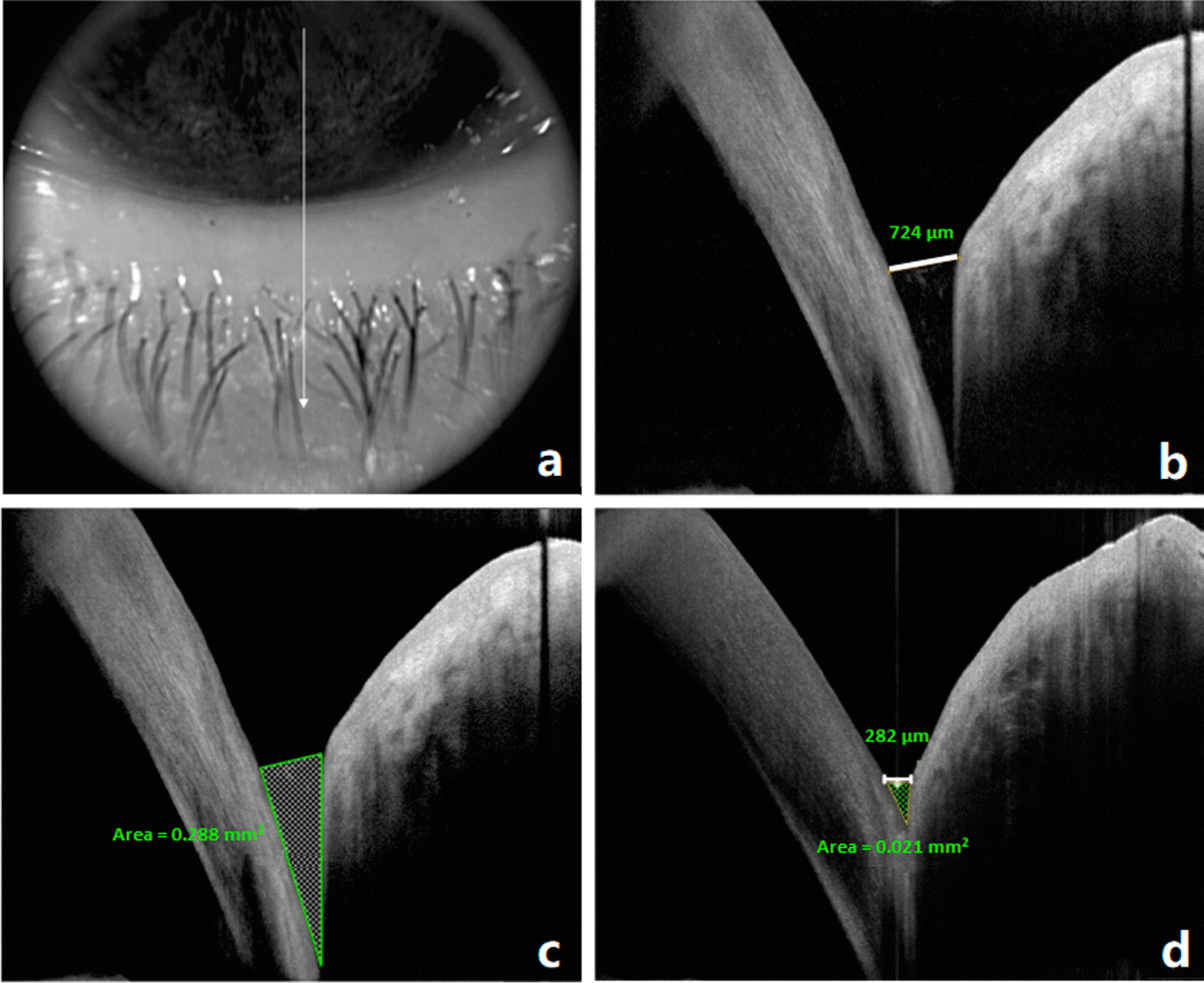
Preoperative and postoperative measurement of tear meniscus height (TMH) and area (TMA). **a** Infrared image showing line scan passing through the junction between the lower eye lid and the inferior cornea at the 6 o’clock location. **b** Preoperative AS-OCT scan showing lower TMH: a line joining upper cornea-meniscus junction to the lower eyelid-meniscus junction (white line, measuring 724 µm). **c** Preoperative AS-OCT scan showing the TMA (hashed triangle, measuring 0.288 mm^2^). **d** Postoperative OCT scan showing TMH (white line 282 µm) and TMA (hashed triangle, 0.021 mm^2^)

Imaging of inferior tear meniscus was done at the lower cornea-lid junction, while the patient was looking straight forward, with the same line scan protocol used for lower punctal evaluation, but vertically oriented and passing through the center of the pupil and crossing the lower limbus at the 6 o’clock position. Tear meniscus height (TMH) in μm and tear meniscus area (TMA) in mm^2^ were measured from the inferior tear meniscus. The OCT images were exported for computer calipers using RTVue software (version 2016.0.0.52, Optovue) for measurements of lower TMH and TMA. The lower TMH was measured using the “measure tool for distances” by measuring the distance from the lower eyelid-meniscus junction to the cornea-meniscus junction. Lower TMA was measured by selecting “measure tool for angles” with the base being the TMH. The two sides of the triangle are the tear-lid contact line and the tear-cornea contact line (Fig. 1).

Objective improvement was evaluated using Munk scale for epiphora grading [12] and patient satisfaction score. A simple questionnaire was adopted and scored from grade 0 to 4 (Additional file 1). The patient was considered satisfied if the score was grade ≥ 3 and dissatisfied if the score < 3.

### Statistical analysis

Statistical analysis was done using the Statistical Package for Social Sciences (SPSS) software version 25. Descriptive statistics were performed for parametric (normally distributed) quantitative data by mean, standard deviation (SD) and minimum and maximum of range, while for non-normally distributed data by median and interquartile range (IQR), and for qualitative data by frequency and percentages. Distribution of the data was tested using the Kolmogorov Smirnov test. Analyses were performed for parametric quantitative data between two occurrences using the paired samples *t*-test and for non-parametric data using the Wilcoxon signed rank test. For parametric quantitative data between two groups, the independent samples *t-*test was used while for non-parametric data, the Mann Whitney test was used. Analyses were performed for qualitative data between two groups using Fisher’s Exact test. Correlation was done using Spearman’s correlation coefficient. The level of significance was taken at *P* < 0.05.

## Results

**Fig. 2:**
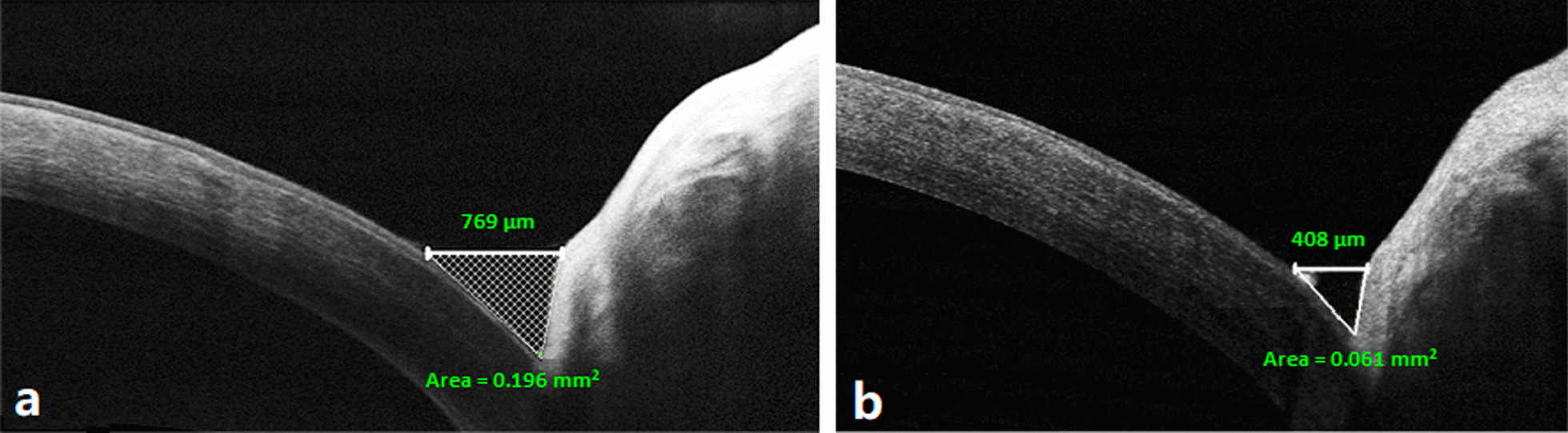
Preoperative and postoperative AS-OCT measurements of tear meniscus height (TMH) and area (TMA). **a** Preoperative scan showing lower TMH (white line, measuring 769 µm) and TMA (hashed triangle, measuring 0.196 mm^2^). **b** Postoperative scan showing TMH 408 µm and TMA 0.061 mm^2^

Fifty-four eyes of 29 patients were included in this study, 6 males (20.7%) and 23 females (79.3%) with mean age of 50.3 ± 4 years (range 43–57 years). Twenty-five patients (86.2%) had bilateral plugs insertion while only 4 patients (13.8%) had unilateral plug insertion for a total of 54 puncta. There was a statistically significant decrease of the postoperative epiphora Munk grade, TMH and TMA compared with the preoperative values (*P* < 0.001; Fig. 2 and Table 1).

**Table 1:** Comparison between pre and postoperative data

Variable	Grade	Preoperative N = 54 (eyes)	Postoperative N = 54 (eyes)	*P* value
TMH (µm)	Range	632–1420	137–959	< 0.001*
	Mean ± SD	936.6 ± 182.3	319.1 ± 204.7	
	Median	875	253	
	IQR	810.3–1019.3	209.8–311.5	
TMA (mm^2^)	Range	0.11–0.73	0.01–0.5	< 0.001*
	Mean ± SD	0.42 ± 0.14	0.07 ± 0.1	
Munk grade	Grade 0	0 (0%)	6 (11.1%)	< 0.001*
	Garde 1	0 (0%)	27 (50%)	
	Grade 2	0 (0%)	14 (25.9%)	
	Grade 3	26 (48.1%)	5 (9.3%)	
	Grade 4	28 (51.9%)	2 (3.7%)	
Postoperative Munk change	Stationary		5 (9.3%)	
	Improved		49 (90.7%)	

Paired samples *t-*test for parametric quantitative data between the two occurrences

Wilcoxon signed rank test for non-parametric quantitative data and ordinal qualitative data between the two occurrences

*TMH* tear meniscus height; *TMA* tear meniscus area; *IQR* interquartile range

^*^Significant level at *P* < 0.05

**Fig. 3:**
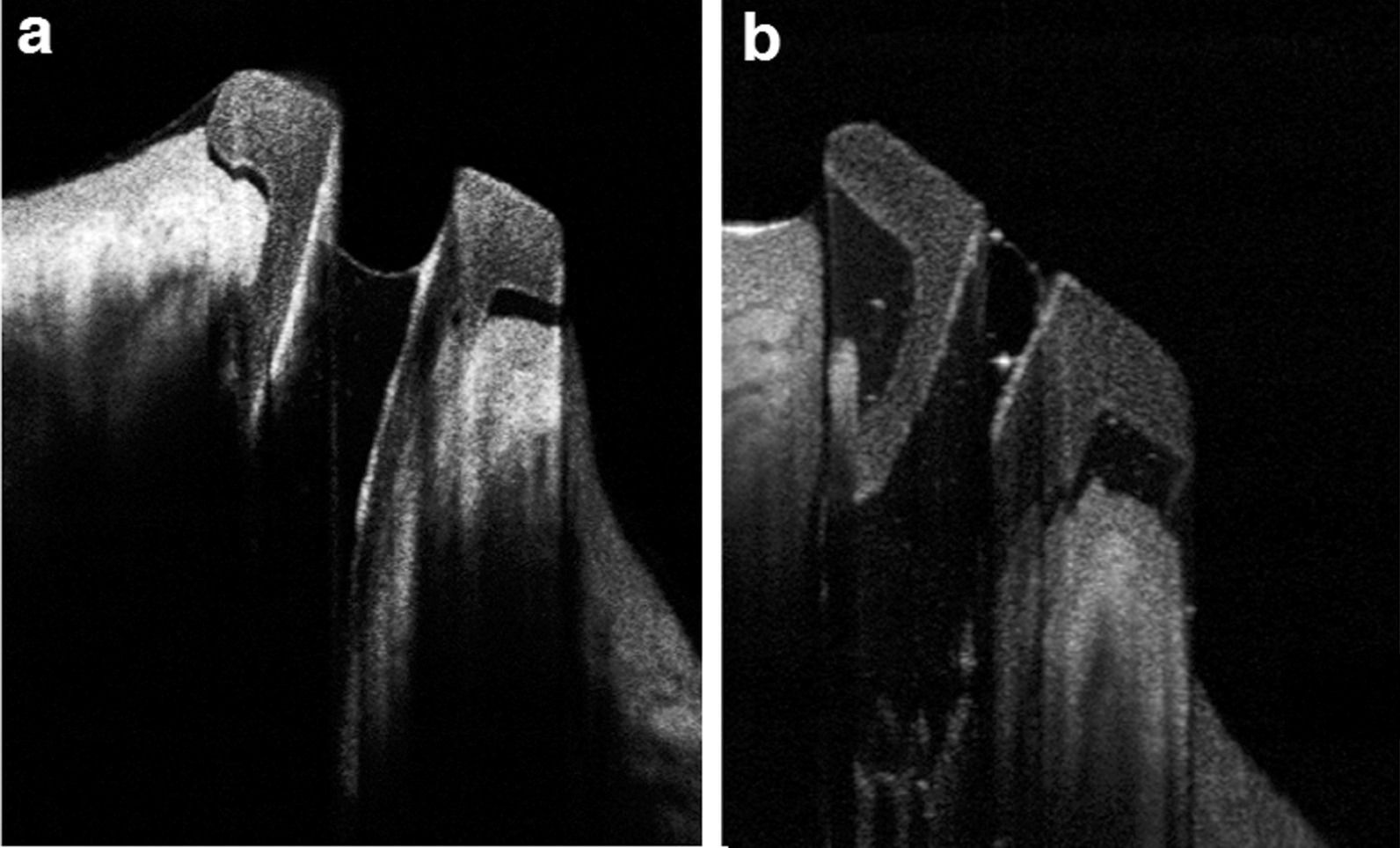
Plug position imaged by AS-OCT. **a** The plug in proper position and fluid level not reaching the top. **b** Rotated plug with debris within its lumen

**Fig. 4:**
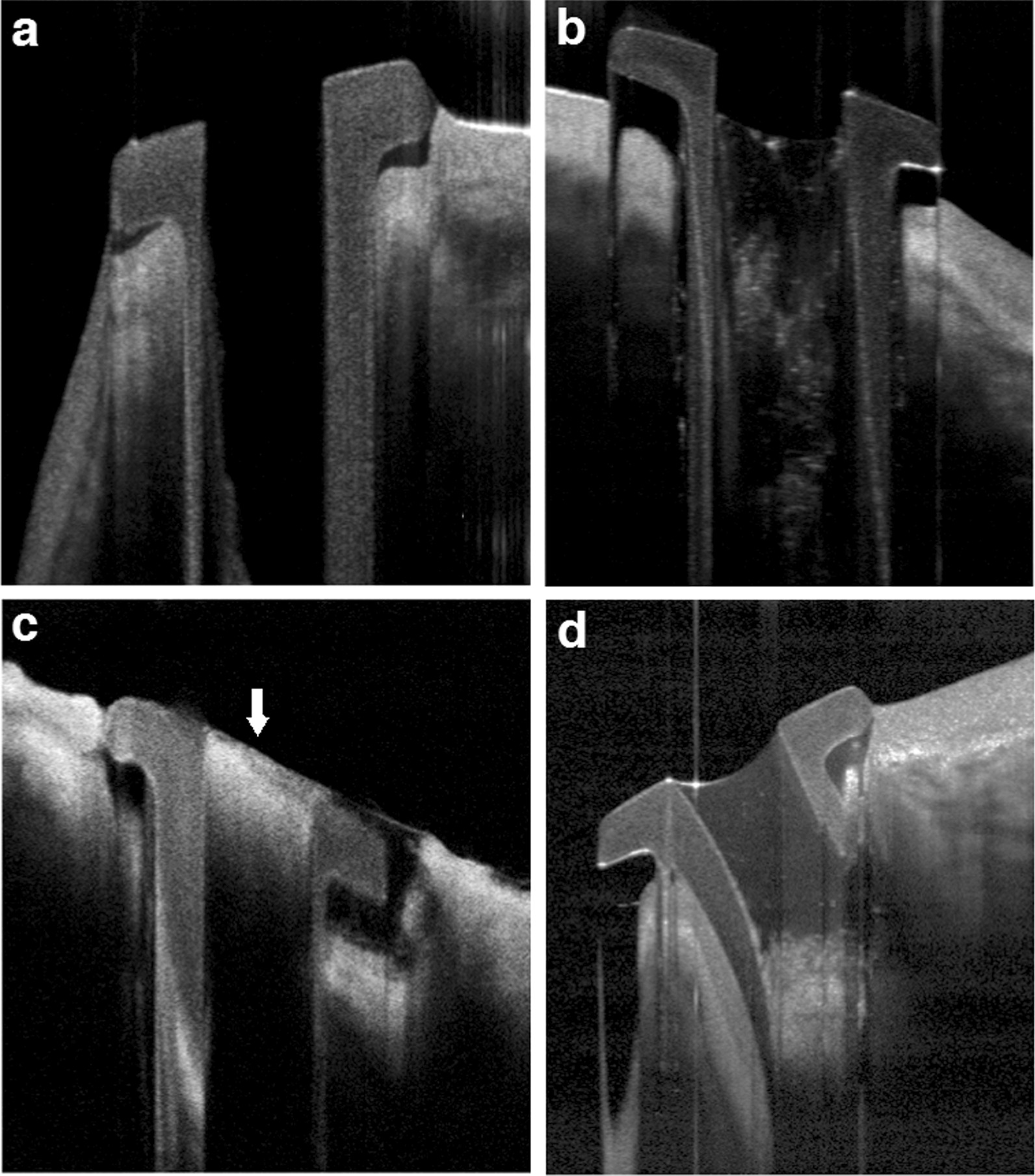
Patency of the plug by AS-OCT. **a** Properly placed patent plug. **b** Partially occluded. **c** Total superficial occlusion (blood clot) demonstrated by arrow and the plug is deeply impacted. **d** Deeply occluded plug and tear fluid reaching the top of the plug

With respect to AS-OCT evaluation of the inserted plugs, all plugs were present in place. However, 48 (88.9%) plugs were found in proper position (Fig. 3a), while 6 (11.1%) were rotated (Fig. 3b). Of the 6 rotated plugs, 3 plugs had patent lumen, while the other 3 plugs were obstructed. The lumen of the plugs was completely patent in 47 (87%) plugs, partially obstructed in 2 (3.7%) plugs and completely occluded in 5 (9.2%) plugs (Fig. 4). Regarding patient satisfaction, 49 patients (90.7%) were satisfied with postoperative outcomes.

There was a statistically significant fair positive correlation between postoperative Munk score and TMH (*r* = 0.385, *P* = 0.004) while its correlation with TMA was statistically non-significant (*r* = 0.221, *P* = 0.108) (Table 2).

**Table 2:** Correlation of postoperative Munk scale with postoperative OCT data

Parameter	Postoperative Munk	
	r	*P* value
Tear meniscus area	0.221	0.108
Tear meniscus height	0.385	0.004*

Non-parametric Spearman’s rho correlation

^*^Significant level at *P* < 0.05

Patients with postoperative patent plugs showed significant postoperative improvement according to the Munk classification, compared with patients with occluded plugs (*P* < 0.001).

No significant difference was found between patients whose plugs were in place and patients with rotated plugs with regards to postoperative change using the Munk classification. In addition, patients who experienced postoperative satisfaction showed significant improvement in Munk classification (*P* < 0.001; Table 3).

**Table 3:** Comparison of postoperative OCT data and patient satisfaction between stationary and improved outcome, assessed by Munk scale

Parameter	Munk change		*P* value
	Stationary	Improved	
	N = 5	N = 49	
Patency			
No	5 (100%)	2 (4.1%)	< 0.001*
Yes	0 (0%)	47 (95.9%)	
In place			
No	1 (20%)	5 (10.2%)	0.459
Yes	4 (80%)	44 (89.8%)	
Filling			
No	0 (0%)	47 (95.9%)	< 0.001*
Yes	5 (100%)	2 (4.1%)	
Abnormal contents			
No	0 (0%)	47 (95.9%)	< 0.001*
Yes	5 (100%)	2 (4.1%)	
Partial occlusion			
No	5 (100%)	47 (95.9%)	1
Yes	0 (0%)	2 (4.1%)	
Postoperative patient's satisfaction			
No	5 (100%)	0 (0%)	< 0.001*
Yes	0 (0%)	49 (100%)	

Independent samples *t-*test for parametric quantitative data between the two groups

Mann Whitney test for non-parametric quantitative data between the two groups

Fisher’s exact test for qualitative data between the two groups

^*^Significant level at *P* < 0.05

## Discussion

Perforated punctal plugs are currently used for treatment of epiphora due to punctal stenosis by maintaining the patency while preserving the sphincter function of the lacrimal punctum after removal of these plugs. Several studies have evaluated the functionality of these plugs [6, 12–14]. AS-OCT is a noninvasive imaging modality that was used in previous studies for the evaluation of normal and stenosed punctal [9, 10] and tear meniscus in normal individuals [11]. In this study, this investigation tool was used to evaluate the position, patency and contents of the perforated punctal plugs during the early postoperative period and these parameters were correlated with the objective postoperative improvement of epiphora using AS-OCT measurements of TMH and TMA and with subjective improvement using postoperative Munk score for epiphora and patient satisfaction. Postoperative evaluation was done 2 weeks after surgery to exclude any possible effects of the early postoperative inflammation on plug position and tear film parameters.

Regarding position, no plug dislodgment or migration was found in this study. In the study of Abd El Ghafar et al. [13] two plugs out of 30 (6.7%) had extruded before 6 months postoperatively versus one plug out of 45 plugs within 2 weeks in the study of Ozgur et al. [14], and also one out of 40 during the 1st month in the study of Tamer et al. [15], who reported additional rotation of four plugs (10%) via the slit lamp examination. The perforated plug was designed to have a shorter medial side compared with the lateral side so that its collar has a higher flange towards the temporal side of the lacrimal papillae and a lower one towards the medial side of the punctum in relation to the lacus lacrimalis to ensure better tear drainage. Rotation of the plug may impair tear drainage. Moreover, the rotated collar may touch the ocular surface causing significant ocular surface irritation that could reflect on the degree of the postoperative epiphora. Significant rotation could be detected during slit lamp examination. However, AS-OCT can demonstrate even subtle degrees of rotation due to its superior ability in imaging the plug collar and the punctal tissue. In our study, AS-OCT demonstrated 48 (88.9%) plugs in proper position while 6 (11.1%) had some degree of rotation without significant correlation between proper plug position and postoperative epiphora. All rotated plugs were repositioned to fit in the proper position.

Regarding patency and contents, perforated plugs could be completely patent, partially, or completely occluded by blood clot or any other debris. Slit lamp examinations can only examine the surface of the plug or the superficial part of its lumen. In some cases, the plug may appear patent with external examination while the patient still has epiphora. This could be because of deep obstruction within the plug or even distal anatomical obstruction through the lacrimal drainage system or functional lacrimal pump disorders which can be differentiated by AS-OCT that can show deeply seated plug obstruction. In the current study, AS-OCT imaging not only evaluated the patency, position, and even fine debris within the lumen of the plug in a very demonstrative manner but also the exact level of obstruction, whether partial or complete and the tear fluid level within the plug. Here, we found that the deeply obstructed plugs have their tear fluid level reaching the top of the plug up to the collar while the patent plugs had tear levels within the lower parts of the plug lumen, or even having the lumen appearing nearly empty. Interestingly, 50% of the rotated plugs had obstructed lumen, indicating that proper plug position enhances tears to flow through its lumen. AS-OCT was a reliable tool in managing cases with postoperative epiphora despite proper plug position and clinically patent plug due to its ability to reveal deep plug occlusion which can be simply managed by irrigating the plug resulting in improvement of epiphora.

There was a statistically significant decrease of the mean TMH measured by AS-OCT from 936.6 ± 182.3 µm before plug insertion to 319.1 ± 204.7 µm postoperatively (*P* < 0.001). Also, TMA significantly decreased from 0.42 ± 0.14 mm^2^ preoperatively to 0.07 ± 0.1 mm^2^ postoperatively (*P* < 0.001). These postoperative values are slightly higher than the results of Raj et al. [11], who used AS-OCT for evaluation of tear meniscus in normal individuals from different age groups. They had mean TMH of 254.2 ± 102.5 µm and mean TMA of 0.02 ± 0.03 mm^2^ in normal individuals in the age group matched with this study (older than the age of 40 years) [11].

Postoperative epiphora was significantly correlated with postoperative TMH more than with TMA. Conversely, Czajkowski et al. [16], reported that the highest correlation with Schirmer test results was found with TMA, followed by TMH. This could be explained based on the different disease nature as they used AS-OCT for quantitative tear evaluation and diagnosis of dry eye syndrome and not epiphora [16].

Finally, postoperative epiphora improved in 49 eyes (90.7%) in comparison to five eyes (9.3%) who experienced no improvement according to Munk’s scale (*P* < 0.001). Patients with more improvement in epiphora reported greater satisfaction.

## Conclusions

In conclusion, AS-OCT is an accurate and valuable tool for postoperative evaluation of perforated punctal plugs, inserted for treatment of epiphora due to punctal stenosis both anatomically and functionally. The ability of AS-OCT to detect even subtle degrees of rotation could be helpful for management just by performing plug repositioning. Moreover, it is superior to clinical examination in detecting any abnormal contents within the narrow lumen of the plugs (0.6 mm) and can explain the presence of epiphora even after properly implanted plugs.

## Supplementary Information

**Additional file 1:** Patient satisfaction score.

## Data Availability

All data generated or analyzed during this study are included in this article.
