# Supplementary material for: Evaluation of implanted perforated lacrimal punctal plugs using anterior segment optical coherence tomography
Source: Eye Vis (Lond). 2021 Oct 3;8:36. doi: 10.1186/s40662-021-00259-x (PMC8487482; doi:10.1186/s40662-021-00259-x)
Supplement: Supplementary file 1 — Additional file 1: Patient satisfaction score. [file 40662_2021_259_MOESM1_ESM.docx]

**Additional file 1: patient satisfaction score:**

1. If there is any improvement of epiphora compared with preoperative state?
2. If this improvement meets the patient expectations?
3. If there is no postoperative irritation or discomfort?
4. If the patient is overall satisfied with procedure?

-For each question if the answer is:

Yes = score (1)

No = score (0)

-The total score is (4)

The patient was considered:

- Satisfied if the score is 3 or more

- Unsatisfied if the score less than 3
